# Event and Comment

**Published:** 1911-12-15

**Authors:** 


### DENTAL REGISTER.

**Vol. LXV. December 15, 1911. No. 12**

### EVENT AND COMMENT.

ORAL SEPSIS. The dental profession was thrown into a spasm of indignant protest last summer by an Editorial comment in *Current Literature* on a paper read by Dr. Wm. Hunter, an English physician, on oral sepsis as a cause of systemic diseases. Almost every dental editor has expressed himself as opposed to the conclusions there drawn. We made our comment on the article as reprinted in a daily newspaper and therefore our attitude was not the same that it is now since we have had an opportunity to read the original article. In many respects, Dr. Hunter takes ground that no intelligent dentist will care to controvert. There can be little doubt that more frequently than we realize there are obscure pathologic dental lesions which may be inimical to general health, and possibly some of them may be active causative influences to specific diseases. Impacted lower third molars are often the cause of local infection with septic results extending to all the contiguous structures; but how many cases of referred pain in the eye, ear, or brain cells may be due to such impactions, with little or no local expressions, we can never know; and it will need no stronger argument than Dr. Hunter has made to convince any thoughtful dentist that the almost innumerable pulps and peridental affections of varying degrees can set up a train of disastrous physical as well as local results. We wish therefore to agree most cordially with Dr. Hunter's main contentions as he states them, but not as his commentators interpret them with such degrading inferences. At the same time we feel that

Dr. Hunter makes a considerable number of statements, which, because of his environment and lack of knowledge of the practice of modern dentistry are wholly wrong. If he possessed a technical knowledge of dentistry he never would have said that, "The prime causal factor in all these processes is the septic infection;—gingivitis, peridentitis, osteitis, and tartar deposits, are the result of septic inflammation and ulceration." Now any dentist knows that septic inflammation ordinarily has no more to do with tartar deposits, gingivitis, or even of peridentitis as causal factors, than it has to do with gray hair or bow legs. These diseases are sometimes concurrent and may fuse together into a homogeneous train of events and yet vary greatly as to their germinal sources.

We can not but feel that Dr. Hunter's article would have been stronger if it had not been somewhat controversial and had not arraigned us as honorable and intelligent practitioners. We are printing the article in this issue,—not complete as published originally,—but with all the essential statements of dental interest and also extracts from two editorials giving two views from our own profession. We trust all our readers who have not had an opportunity to read the original will take the time and carefully read this presentation.

THE TAGGART PATENT SUIT DISMISSED. A Washington paper contains the information that chief Justice Clabaugh of the Supreme Court of the District of Columbia has dismissed the suit brought by Dr. Taggart to enjoin Dr. Boynton, of Washington, from infringing his rights of patent on a method of casting gold inlays. The costs in the case were assessed on Dr. Taggart. The paper states that when the case was called Taggart's attorneys did not press their suit and the order of dismissal was entered. Whether this means the end of litigation or not we don't know, but if Dr. Taggart has dropped the case because he does not want to coerce the profession it will now be time for the profession to make good some statements that have been made in regard to recompensing Taggart professionally.

